# Management of a Dropped Autologous Skin Graft

**Published:** 2014-09-27

**Authors:** Ronald M. Brooks, Donald R. Herdt, Morton Kasdan

**Affiliations:** Robley Rex VA Medical Center, University of Louisville, Louisville, Ky

**Keywords:** graft, contamination, autologous, infection, flap

**Figure:**
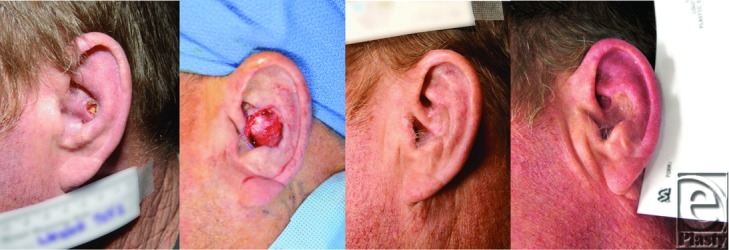


## DESCRIPTION

A 67-year-old man underwent full-thickness skin graft reconstruction of an ear defect following skin cancer excision. The graft was dropped on the floor during preparation. Following decontamination, it was subsequently used to complete the reconstruction, with full graft take and no evidence of infection after 4 months of follow-up.

## QUESTIONS

1. **How can one prevent graft contamination?**
2. **What is the preferred method for management of a contaminated graft?**
3. **Is there an antibacterial solution with superior efficacy?**
4. **How should one approach medical error disclosure?**

## DISCUSSION

Although likely underreported, graft contamination appears relatively a commonplace. In a survey, 70% (157/223) of responding plastic surgeons reported a graft contamination experience, and more than 70% of these surgeons reported multiple occurences.^1^ In a survey of orthopedic surgeons, 25% (49/196) reported contaminating an anterior cruciate ligament graft.^2^ Contamination prevention should involve minimizing and mitigating human error. Recommendations include notifying the ancillary staff of graft usage, avoiding manipulation of the graft outside of the sterile field, placing the graft inside a covered container away from trafficked areas, and minimizing the number of tissue hand offs. In our patient, the graft was dropped on the floor during preparation near the back table. In our case, we could have avoided contamination by simply preparing the graft immediately overlying the sterile field.

Similar to our case, the majority of reported graft contaminations are a result of falling on the floor. Unfortunately, there is no consensus on a management protocol for dropped grafts, but the preferred method among plastic surgeons appears to be decontamination and completion of grafting, with povidone-iodine solution being the most popular decontaminant.^1^ Other decontamination solutions reported in the literature include chlorhexidine gluconate, antibiotic mixtures, sodium hypochlorite, and hydrogen peroxide. In our patient, decontamination was performed with povidone-iodine followed by normal saline lavage.

The majority of available literature regarding graft decontamination focuses on bone and tendon grafts, with relatively few studies looking specifically at contaminated skin grafts. Results are largely inconsistent, but many studies recommend decontamination with either chlorhexidine gluconate^1^^,^^3^ or povidone-iodine.^4^^,^^5^ It is worth noting that povidone-iodine has been shown to be toxic to skin fibroblasts in vitro,^6^ decreasing its appeal for use on skin and soft tissue. Regardless of the decontamination method used, only 1.9% (3/157) of surveyed plastic surgeons reported infection after usage of a contaminated graft.^1^ This actually compares favorably to the 1.51% reported infection rate for low-risk clean skin graft surgery.^7^ This suggests that as long as grafts are decontaminated in some fashion, their subsequent use is safe with low morbidity.

After accidental contamination, full disclosure to the patient is an ethical imperative. Surprisingly, in the survey by Centeno et al, 60% of the surveyed surgeons did not disclose the incident and only 20% informed the patient/family postoperatively. In addition, full disclosure is also likely in the best interest of the physician, as nondisclosure is a frequently cited reason for malpractice law suits.^8^ In a thorough article on disclosure of medical error, Gallagher et al^8^ recommended that the minimal information include “(1) an explicit statement that an error occurred; (2) a basic description of what the error was, why the error happened, and how recurrence will be prevented; and (3) an apology.”

When deciding whether or not to salvage a contaminated graft, the surgeon must take into consideration the individual risk factors of the patient, blood supply to the graft, donor tissue type, and possible alternatives reconstructive options. While there is a dearth of literature on the management of contaminated autologous skin grafts, survey data suggests that it can be salvaged without significant morbidity.
